# Cutaneous Myiasis Presenting as Painful Leg Nodules Following a Fishing Trip to El Salvador: A Case Report

**DOI:** 10.7759/cureus.89436

**Published:** 2025-08-05

**Authors:** Khwaja S Hasan, John F Dunn, James A Swaby, Richard R Jahan-Tigh

**Affiliations:** 1 Biomedical Sciences, University of Waterloo, Waterloo, CAN; 2 Dermatology, University of Texas Health Science Center at Houston McGovern Medical School, Houston, USA; 3 Medical Entomology, United States Air Force, San Antonio, USA

**Keywords:** botfly, cutaneous furuncular myiasis, dermatobia hominis, el salvador, larva

## Abstract

We report the case of a 50-year-old Hispanic man with recent travel to El Salvador, who developed two painful nodules on his right lower extremity following a fishing trip. Upon medical evaluation, the nodules were diagnosed as cutaneous myiasis, likely caused by *Dermatobia hominis*. Videos demonstrating a minimally traumatic larval extraction technique are included. This case underscores the importance of considering myiasis in the differential diagnosis of painful skin nodules in returning travelers and highlights an effective removal method.

## Introduction

Furuncular myiasis is a cutaneous parasitic infection caused by the larvae of certain flies in the Diptera order, most commonly affecting individuals at risk for arthropod exposure, such as outdoor workers, hobbyists, and travelers [1]. Clinically, it presents as a raised, tender nodule with a central punctum, which may drain serosanguinous or pustular fluid. The differential diagnosis includes arthropod bites or stings, folliculitis, and bacterial abscesses. Patients typically experience localized pain and pruritus as the larva feeds and grows within the skin [1]. *Dermatobia hominis*, commonly known as the human botfly, is a leading cause of furuncular myiasis and is endemic to Central and South America and other tropical regions [1,2]. The life cycle begins when a gravid female fly lays eggs onto a vector, typically a mosquito [1]. During the mosquito’s blood meal, the eggs are transferred to the human host’s skin, where they hatch, burrow, and mature over several weeks before emerging [1]. Although secondary bacterial infection is possible, it remains relatively uncommon [3]. Healing generally occurs with or without residual scarring [1]. This case underscores the importance of recognizing myiasis in returning travelers from endemic areas and highlights the value of preventative education for individuals planning travel to such regions.

## Case presentation

A 50-year-old Hispanic man presented to the dermatology clinic in July 2024 for evaluation of two slowly enlarging, intermittently painful nodules with occasional bleeding on the right proximal pretibial region. The patient reported that both lesions appeared simultaneously approximately two weeks earlier. He noted that pain and bleeding were exacerbated by physical activity and kneeling. His medical history was notable only for mild hyperlipidemia, managed with simvastatin. He denied prior similar lesions. Six weeks before presentation, the patient had traveled to El Salvador, where he engaged in swimming in a lake and fishing. He did not recall any rashes or arthropod bites during his stay. He had applied topical neomycin-polymyxin B-bacitracin ointment without improvement.

On physical examination, two tender, 2 cm, slightly erythematous nodules were observed on the medial and posteromedial aspects of the right lower leg, each with a small central punctum. Notably, intermittent movement was visible within the nodules (Video 1). Local anesthesia with 1% lidocaine with epinephrine was administered, and larvae were extracted from each lesion using forceps. The extraction procedure is shown in Video 2.

**Video 1:** Preparation of the extraction site

**Video 2:** Extraction of the larva

**Figure 1:**
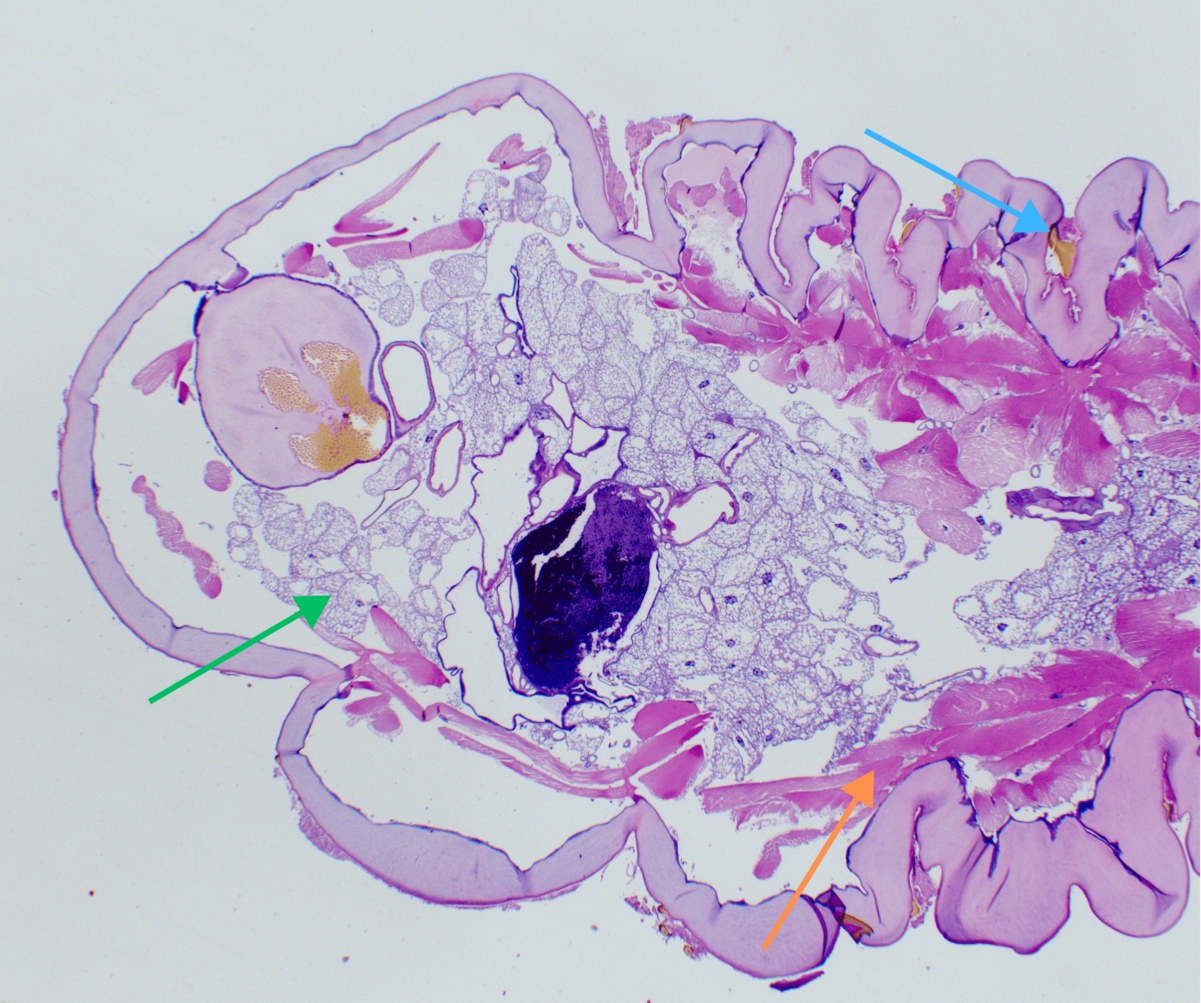
Hematoxylin and eosin-stained sections of the larva (200x magnification) The orange arrow highlights the striated musculature, the green arrow points to the salivary glands, and the blue arrow highlights the cuticular spines.

One of the extracted larvae was preserved in 10% phosphate-buffered formalin and measured 1.9 × 0.5 × 0.5 cm. The specimen was bisected, processed, and stained with hematoxylin and eosin (H&E). Key anatomical features of the arthropod are annotated in Figure 1. Following extraction, the wound sites were managed by allowing closure through secondary intention.

## Discussion

While most cases of New World furuncular myiasis occur in the tropical regions of South and Central America, rising global tourism has been associated with an increase in reported cases within the United States [2]. Additionally, increasing global temperatures have expanded the geographic range of arthropod vectors responsible for transmitting various diseases [4]. A notable case from Fort Walton Beach, Florida, suggests that indigenous transmission of myiasis is possible in the United States, even in the absence of recent travel history [5]. Clinical manifestations of *D. hominis* have also been documented in various anatomical regions, including the eyelid, canthus, scalp, and urogenital areas [1,5,6]. Although histological analysis in the present case could not definitively confirm the taxonomy, the arthropod's anatomical features were consistent with *D. hominis*.

Typically, *D. hominis* produces a single lesion harboring a single larva and accounts for most cases of myiasis in exposed areas [1]. However, other species of parasitic flies also contribute to human disease, with varying clinical presentations. *Cordylobia anthropophaga*, prevalent in Africa, often causes multiple lesions on covered areas, especially during the rainy season [1]. Children are at higher risk, and the condition may provoke intense inflammatory reactions [1]. In contrast, *Wohlfahrtia vigil*, though rare, has been reported in North America and may cause multiple lesions containing 1-5 larvae each [1]. These lesions often appear during warmer months and may be preceded by a plaque [1].

Various methods have been successfully employed for larval extraction in cases of furuncular myiasis. Occlusive agents such as petroleum jelly can suffocate the larva by blocking its breathing pore [1]. Oral ivermectin, administered at 200 μg/kg body weight, has also been reported to facilitate larval removal [6]. Surgical excision under local anesthesia remains a common and effective approach [1]. Preventive strategies include the use of insect repellents, mosquito netting, and protective clothing to minimize exposure to vector insects [1]. If left untreated, myiasis can lead to complications such as secondary bacterial infection, regional lymphadenopathy, systemic symptoms including fever, severe pain, and the development of indurated plaques or cellulitis [1].

## Conclusions

With the rise in international travel, the incidence of travel-associated myiasis is expected to increase. This underscores the need for clinicians to maintain a high index of suspicion when evaluating skin lesions in patients returning from endemic regions. Fortunately, multiple safe, simple, and cost-effective methods are available for larval removal, and most patients recover fully. Enhancing public and clinical awareness of cutaneous furuncular myiasis can lead to earlier diagnosis and timely intervention. Additionally, employing preventive measures, such as insect repellents, protective clothing, and mosquito netting, can significantly reduce the risk of infection.
